# Optimization of Phenolic Compounds Recovery from Pistachio Hull Using Accelerated Solvent Extraction

**DOI:** 10.3390/antiox15050558

**Published:** 2026-04-28

**Authors:** Ana I. Paniagua-García, Lucía Gómez-González, Silvia González-Rojo, Rebeca Díez-Antolínez

**Affiliations:** 1 Centre of Biofuels and Bioproducts, Agricultural Technological Institute of Castilla y León, Villarejo de Órbigo, 24358 Leon, Spaindieantre@itacyl.es (R.D.-A.); 2 Department of Applied Chemistry and Physics, University of León, Campus de Vegazana s/n, 24071 Leon, Spain; sgonr@unileon.es

**Keywords:** pistachio hull, accelerated solvent extraction, phenolic compounds, antioxidant activity, response surface methodology

## Abstract

Pistachio hull (PH) is the largest by-product of the pistachio industry. It contains high levels of phenolic compounds, which have antioxidant properties and promote health. However, PH can accumulate during harvesting, potentially resulting in environmental pollution. This study aimed to optimize the operational conditions for conventional solvent extraction (CSE) and accelerated solvent extraction (ASE) of phenolic compounds from PH using response surface methodology (RSM). The extracts obtained under optimal conditions for the two extraction techniques were compared. The highest values of phenolic recovery (9.92 ± 0.09 g gallic acid equivalent (GAE)/100 g dried matter (DM)) and radical-scavenging activity for α,α-diphenyl-β-picrylhydrazyl (DPPH) (IC50 = 0.16 ± 0.00 mg/mL) were achieved by the extract obtained by ASE (23% ethanol in water, 180 °C, 15 min). Regarding individual phenolic compounds, gallic acid accounted for 35.7–48.1% of the total phenolic compounds contained in the PH extracts, followed by 3,4-dihydroxybenzoic acid, 4-hydroxybenzoic and 2,5-dihydroxybenzoic acid. The findings of this study demonstrate for the first time that PH can be valorized by ASE using eco-friendly solvents, obtaining extracts with a high phenolic content, reducing waste and promoting the bioeconomy development.

## 1. Introduction

The pistachio (*Pistacia vera* L.) is a high-value crop that originated in the arid regions of Central Asia before spreading throughout the Middle East and the Mediterranean area [1,2]. It is a member of the Anacardiaceae family [1]. In 2022, approximately 1,026,803 tonnes of pistachio in shell were produced worldwide [3]. The processing of whole pistachios results in the generation of significant amounts of by-products, including pistachio hull (PH), leaves, clusters, residual kernel and hard woody shell, representing over 60% of the total production, with the PH being the predominant by-product [4]. It is imperative that pistachios undergo processing within 24 h of harvesting to prevent hull-trapped moisture, which can cause staining of the pistachio hard shell [1]. The PH is separated from the hard-woody shell and accumulated in large volumes and if not processed, this can lead to environmental problems and challenges in waste management [5]. PH is frequently intermingled with soil, yet its elevated water content renders it susceptible to microbial contamination and fermentation, which may ultimately lead to crop deterioration [5]. In certain regions, PH is utilized as a constituent of animal feed. Nevertheless, the elevated level of phenolic compounds it contains has the potential to induce nutritional imbalances in livestock [1]. Recently, researchers have directed attention to PH due to its high content of phenolics, primarily flavonoids and tannins, including gallic acid, quercetin, gallotannins, galloyl derivatives and proanthocyanidins [1,6]. Additionally, studies have demonstrated that PH extracts possess antioxidant, anti-inflammatory, antimutagenic, cytoprotective and antimicrobial properties [5].

In addition to cultivar, soil condition, climate, geographical location and processing methods, the variability in functional compounds and extraction yield is also dependent on the extraction method selected and the choice of appropriate solvent [1]. In this context, Accelerated Solvent Extraction (ASE) is a highly efficient and sustainable extraction method that is also environmentally friendly [7]. This method offers attractive options for industrial processes [7,8]. The ASE process reduces the use of solvents and water while facilitating the valorization of the residues obtained after extraction [9]. ASE is based on the use of temperatures above the solvent boiling point but bellow the critical point. Consequently, the extraction rate increases while maintaining the solvents in a liquid state through the application of high pressures [10]. ASE has several advantages over conventional solvent extraction (CSE). These include the use of smaller volumes of extraction solvents, an increase in extraction yield and the performance of automatic equipment that is easy to use, as well as can increase the reproducibility and quality of the obtained extracts [7,8]. Furthermore, this technique can be scaled up to valorize natural by-products [8]. On the other hand, the main limitations of ASE are the low selectivity and dilution of the extracted compounds, the high cost of equipment and the rigorous optimization of extraction variables [7,11].

The choice of extraction solvent is another key factor in achieving the maximum yield of bioactive compounds in the final extract [8]. Phenolic compounds are predominantly polar, making water and alcohols effective solvents for achieving high extraction yields [1]. To develop more environmentally friendly processes, it would be preferable to use “generally recognized as safe” (GRAS) solvents such as water and ethanol [8]. According to the literature, water and hydroalcoholic mixtures have been demonstrated to yield high phenolic extraction recoveries from plant resources, although contradictory results have been reported [1,5,6]. Furthermore, research has demonstrated that temperature and extraction time are key factors influencing the recovery of phenolic compounds [12]. Consequently, an increase in extraction temperature typically results in an increased extraction yield. However, the combination of elevated temperatures and prolonged extraction times can facilitate the degradation of the phenolic compounds, leading to a reduction in their concentration and antioxidant properties [12]. Therefore, it is crucial to optimize the extraction variables for each biomass type and extraction technique to maximize the recovery of phenolic compounds, as each biomass has a unique profile of compounds. According to the literature, some studies have been conducted to optimize the extraction variables of phenolics from pistachio by-products using different extraction techniques. These include works carried out on biowastes from pistachio processing using Ultrasound-Assisted Extraction (UAE) and CSE with stirring [12], pistachio kernel through subcritical fluid extraction [13] and pistachio shells using Microwave-Assisted Extraction (MAE) [14]. Furthermore, several studies have documented the extraction of phenolics from PH using CSE with magnetic stirring [2,15], enzyme-assisted aqueous extraction [16], MAE [5,17] and UAE [17]. Moreover, a number of studies have previously reported on the extraction of phenolics using ASE on various natural resources, including chaga, grape skins and seeds, chestnut bur, apple pomace and strawberry leaves [8,18,19,20,21]. These studies demonstrate the high extraction yield achieved by the application of this technique. Therefore, Alhallaf et al. [18] reported the recovery of 3.85 g gallic acid equivalents (GAE)/100 g dry matter (DM) of phenolics from chaga by ASE (66% of ethanol, at 170 °C for 7 min). Conversely, conventional extraction methods such as maceration extraction (70% ethanol, at room temperature for 48 h), reflux extraction (70% ethanol, at 70 °C for 3 h) and Soxhlet extraction (70% ethanol, at temperature adjusted to maintain a low and constant boil for 48 h) yielded 0.31, 0.86 and 0.66 g GAE/100 g DM, respectively. In addition, Paniagua-García et al. [8] achieved a phenolic recovery of 8.37 g GAE/100 g DM from chestnut bur by ASE under optimal conditions (31.3% ethanol, 180 °C for 9 min). By contrast, through the application of the optimized conditions of CSE (31.3% ethanol, 80 °C for 2.22 h with magnetic stirring) the phenolic recovery was 5.70 g GAE/100 g DM. In addition, the study reported by Terpinc et al. [21] demonstrated higher phenolic recovery from strawberry leaves using ASE (8.03 g GAE/100 g DM: 150 °C for 5 min) compared to MAE (6.31 g GAE/100 g DM: 80 °C for 5 min). Consequently, the preceding studies have demonstrated the efficacy of ASE in the recovery of phenolic compounds from agri-food by-products. However, to the best of our knowledge, no studies have been found in the literature focusing on the extraction of bioactive compounds from pistachio by-products using ASE.

The present study focused on evaluating the efficiency of ASE for extracting phenolic compounds from PH. The objective of the research was to optimize the extraction parameters of ASE and CSE, using water and hydroalcoholic mixtures, with the aim of maximizing the total phenolic recovery from PH. Following the verification of the optimal extraction conditions, a comparative analysis was conducted on the effectiveness of both extraction techniques and solvents in terms of phenolic recovery from PH. In addition, the composition of the optimal PH extracts and their phenolic composition was examined. Furthermore, the efficiency of 2,2-diphenyl-1-picrylhydrazine (DPPH) and ferric reducing antioxidant power (FRAP) assays of all the optimal extracts obtained from PH under optimal conditions was investigated. To the best of our knowledge, this is the first report of phenolic extraction from PH using ASE. While most industrial-scale processes for extracting phenolics from plants are carried out using CSE methods involving temperature-controlled stirred tanks, the scaling up of alternative extraction techniques is currently being studied [10]. In this context, this study represents a foundation step in the ongoing effort to enhance the industrial process of phenolic extraction from PH.

## 2. Materials and Methods

### 2.1. Reagents and Biomasses

The Folin-Ciocalteau reagent was obtained from Scharlab (Barcelona, Spain). Analytical-grade 2,2-diphenyl-1-picrylhydrazyl (DPPH), 2,4,6-Tris(2-pyridyl)-s-triazine (TPTZ), cinchonine hemisulfate salt and the following analytical standards: 3-hydroxybenzoic acid, 4-hydroxybenzoic acid, 2,5-dihydroxybenzoic acid, 3,4-dihydroxybenzoic acid, gallic acid monohydrate, syringic acid, vanillic acid, caffeic acid, *p*-coumaric acid, ferulic acid, vanillin, syringaldehyde, ascorbic acid and (+)-catechin hydrate were provided by Sigma-Aldrich (Steinheim, Germany). Analytical-grade absolute ethanol (≥99.8%, *v*/*v*) was purchased from Panreac (Castellar del Vallés, Spain). Other chemicals were of analytical grade and obtained from Thermo Fisher Scientific (Waltham, MA, USA). Deionized water (resistivity > 18 MΩ/cm) was produced by using a Milli-Q ultrapure system from Millipore (Burlington, MA, USA).

PH was supplied by Alternativas Agroforestales ESLA S.L. (Valladolid, Spain) and collected in November 2022. The biomass was dried in an oven at 45 °C for 48 h, ground in a cutting mill SM100 Comfort (Retsch GmbH, Haan, Germany), sieved to a size of 0.5–1.0 mm and stored at room temperature in airtight containers until use. The compositional analysis of the PH (dry basis) was: 10.64 ± 0.33% cellulose, 10.30 ± 0.32% hemicellulose, 20.86 ± 0.16% Klason lignin, 12.17 ± 0.69% protein, 7.27 ± 0.24 fat and 0.54 ± 0.03% ash. The total monomeric sugar content was 24.26 ± 0.72%. The chemical characterization was performed according to Paniagua-García et al. [22].

### 2.2. Phenolic Compounds Extraction from Pistachio Hull

#### 2.2.1. Conventional Solvent Extraction

All CSE experiments were performed by weighing 1 g of crushed PH into 100 mL Erlenmeyer flasks and extracting with 50 mL of water or hydroalcoholic mixtures with varying percentages of ethanol (Section 2.3.1). The flasks were stoppered with a rubber septum and magnetically stirred (200 rpm) at the temperature and for the time specified in the experimental design (Section 2.3.1). After solid–liquid extraction, the mixture was allowed to cool, and the liquid extract was recovered by vacuum filtration using a Büchner funnel with cellulose filters (20–25 µm, Model 1238, Filter Lab, Barcelona, Spain). The spent solid retained on the cellulose filter was then washed with two 20 mL aliquots of fresh extraction solvent, which were collected and added to the liquid extract. Finally, the volume of each extract was adjusted to 100 mL in a volumetric flask with fresh solvent and stored at −20 °C until analysis.

#### 2.2.2. Accelerated Solvent Extraction

The ASE process was carried out using a Dionex ASE 350 (Thermo Fisher Scientific) equipped with a solvent controller to ensure precise adjustment of the solvent ratios. In summary, 2 g of diatomaceous earth and 1 g of ground PH were mixed and then packed into a 34 mL stainless steel extraction cell. The cells were fitted with a stainless steel frit and a cellulose filter (Thermo Fisher Scientific) at the bottom to prevent suspended particles from accumulating in the vial [8]. To carry out the extraction process, water or hydroalcoholic mixtures were used, applying different temperatures for varying times depending on the experimental design (Section 2.3.2). During the first step, the cell containing the sample was filled with the extraction solvent. The cell was then pressurized to 1500 psi (10.3 MPa) and heated to the desired extraction temperature. The static time was set to 0 min. The static phase of the extraction was carried out with all the valves closed during the defined extraction time, maintaining constant pressure and temperature. Subsequently, the cell and tubing were rinsed with 60% of the cell volume of fresh extraction solvent and purged with nitrogen for 90 s to remove any residual solvent. The resulting extracts were collected in a bottle, and the unit was pressure released. The system was thoroughly rinsed between successive extractions to prevent transfer from one experiment to the next. The resulting extracts were adjusted to exactly 100 mL in a volumetric flask with fresh solvent and stored at −20 °C until analysis.

### 2.3. Optimization of Phenolic Extraction Conditions

The CSE and ASE variables were optimized using Response Surface Methodology (RSM) to maximize the extraction efficiency of phenolic compounds from PH.

#### 2.3.1. Optimization of Conventional Solvent Extraction

To optimize the extraction of phenolics from PH by CSE, two central composite rotatable designs (CCRD), were employed. One design was used for water-based solvent extraction, while the other was used for extraction using an ethanol and water mixture. Regarding the extraction carried out with water, a two-variable CCRD was used to determine the optimum combinations of temperature (°C) and extraction time (min) to achieve maximum phenolic recovery. The temperature ranged from 50 to 95 °C and the stirring time ranged from 30 to 120 min. The experimental design consisted of 13 trials. For the extraction performed with a mixture of ethanol–water, a three-variable CCRD was designed to calculate the optimal combinations of temperature (°C), stirring time (min) and ethanol concentration (%, *v*/*v*) to maximize phenolic extraction. Based on preliminary experiments, temperature was evaluated between 40 and 80 °C, the stirring time between 30 and 120 min and the ethanol concentration between 0 and 100%. The experimental design resulted in 20 trials. All the experiments were performed in a single replicate.

#### 2.3.2. Optimization of Accelerated Solvent Extraction

In order to optimize the operating variables of the ASE to maximize phenolic recovery from PH, two CCRDs were performed. A two-variable CCRD (13 trials) was used to determine the optimal combinations of temperature (°C) and heating time (min) for water-based ASE. The temperature range to be evaluated was selected based on preliminary experiments and considering the potential degradation of phenolic compounds at high temperatures [10]. Consequently, temperatures ranging from 50 to 180 °C were examined. Furthermore, the heating time was evaluated from 2 to 30 min. Additionally, a three-variable CCRD (20 trials) was designed to optimize ASE performed with a mixture of ethanol and water. The experimental design was planned to determine the optimal combination of temperature (°C), heating time (min) and ethanol concentration (%, *v*/*v*). The temperature was evaluated between 50 and 180 °C, the stirring time between 2 and 30 min and the alcohol concentration between 0 and 100%. Each extraction trial was performed in a single replicate.

For all CCRDs, including those for CSE and ASE, the output results (phenolic recovery) were fitted to second-order polynomial equations, one for each type of extraction and solvent, and the regression coefficients were determined using analysis of variance (ANOVA). The optimal extraction variables were calculated from the models. Additional CSE and ASE experiments were performed in triplicate with each extraction solvent to validate the regression models under the predicted optimal conditions.

### 2.4. Analysis of the Pistachio Hull Extracts

The total phenolic content (TPC) of the PH extracts was determined using the Folin–Ciocalteu reagent and following the procedure described by Paniagua-García et al. [8]. The results were expressed as g GAE/100 g DM.

The total flavonoid content (TFC) was measured using an AlCl_3_·6H_2_O solution [8], and the results were expressed as g catechin equivalents (CE)/100 g DM.

The total tannin content (TTC), total hydrolyzable tannin content (THTC) and total proanthocyanidin content (TPrC) were determined by precipitating the tannins with a cinchonine hemisulphate solution and using the Folin–Ciocalteu reagent [8]. The TTC was calculated as the difference between the TPC of the initial PH extract and the phenolic content of the non-tannin supernatant [8]. Subsequently, after dissolving the tannin precipitate with ethanol–water (1:1, *v*/*v*), the TPC measurement of the supernatant represents the THTC, and the TPrC was calculated as the difference between the TTC and the THTC [8]. The results were expressed as g GAE/100 g DM.

The concentration of 12 individual phenolic compounds in the PH extracts was determined after hydrolysis of the gallotannins with an HCl solution [8]. Subsequently, the concentration of the compounds was measured using an Agilent 1260 Infinity II Prime LC system (Agilent Technologies, Santa Clara, CA, USA), which was equipped with a diode array detector (DAD) and an analytical Waters Resolve C18 (300 mm × 3.9 mm, 5 µm) column (Waters Corporation, Milford, MA, USA). The analytical method has been described elsewhere [22]. The results were expressed as mg/g DM.

The ferric reducing antioxidant power (FRAP) of the PH extracts was evaluated using the 2,4,6-tris(2-pyridyl)-s-triazine (TPTZ) probe [8]. The results were expressed as g ascorbic acid equivalents (AAE)/100 g DM.

The antiradical-scavenging activity (RSA) of the extracts was determined using a 2,2-diphenyl-1-picrylhydrazyl (DPPH) radical probe in a stoichiometric assay [8]. The RSA was calculated as follows: RSA (%) = 100 × [(A0 − AS)/A0], where A0 is the absorbance of the control solution and AS is the absorbance of the diluted sample. The extract concentration that provides the maximum radical-scavenging activity (C RSA_max_) and the extract concentration that exhibits 50% of the radical-scavenging activity (IC50) were calculated by plotting RSA (%) versus extract concentration. The results were expressed as mg DM/mL required to achieve C RSA_max_ and IC50. Catechin and ascorbic acid were used as standards.

All analyses were performed in triplicate.

### 2.5. Statistical Analysis

Experimental designs were analyzed using the CCRD-based RSM with Minitab 16 software (Minitab, State Collage, PA, USA). Second-order polynomial models were fitted to determine the relationship between the independent variables (temperature, extraction time and, where applicable, ethanol concentration) and the response variable (TPC) for each CCRD. The adequacy of each model was assessed using ANOVA to evaluate the overall significance of the model (*p* value < 0.05) and the R^2^ to estimate the goodness of fit. Model assumptions were verified prior to ANOVA: the normality of residuals was checked using Shapiro–Wilk test, and the homogeneity of variances was confirmed by Levene’s tests. In addition, residuals were visually inspected using normal probability plots and residuals-versus-fitted plots to detect possible deviations or outliers.

Contour plots and response surface plots were generated to visualize the main interaction effects of the independent variables on phenolic recovery and to identify the optimum extraction conditions. The predicted optimal values were experimentally validated and calculated and observed responses were compared to confirm model adequacy.

For PH extracts obtained under optimized conditions, results were expressed as mean values ± standard deviation (n = 3). Differences among extraction treatments were assessed using one-way ANOVA followed by Tukey’s HSD test at a significance threshold of *p* < 0.05 (Statistix 10.0 Analytical Software, Tallahassee, FL, USA).

## 3. Results and Discussion

### 3.1. Optimization of Conventional Solvent Extraction Conditions and Verification of the Models

To optimize phenolic recovery from PH using CSE, a two-variable CCRD (temperature and stirring time) and a three-variable CCRD (temperature, stirring time and ethanol concentration) were performed using water (Table 1) and a mixture of ethanol and water (Table 2). Experiments involving water produced variable phenolic recoveries, ranging from 5.76 to 7.38 g GAE/100 g DM. Furthermore, experiments conducted with an ethanol and water mixture showed phenolic recovery values ranging from 2.28 to 8.05 g GAE/100 g DM.

To analyze the experimental results and evaluate the effects of the independent variables and their possible interactions on CSE, two ANOVAs were performed, one for each type of extraction solvent. According to the calculated quadratic model, 89.25% of the variation in the phenolic recovery using water as solvent could be explained (Table S1). Furthermore, for the CSE of PH using an ethanol and water mixture as extraction solvent, the corresponding mathematical model explained 90.55% of the variation in the phenolic recovery (Table S2). Therefore, the calculated models for the CSE carried out with the two types of extraction solvents indicated good fits to the experimental data. In addition, the quadratic models predicted by both extraction solvents demonstrated significance at the 95% confidence level with *p*-values less than 0.05 (0.003 and 0.000, respectively) (Tables S3 and S4). In the water-based CSE, temperature exhibited a more significant impact on phenolic recovery values compared to stirring time. Moreover, the impact of temperature on the analyzed response variable was found to be statistically significant (*p* < 0.05), while the effect of stirring time was not statistically significant (*p* > 0.05). In the case of CSE performed with a mixture of ethanol and water, only the effect of the ethanol concentration was statistically significant (*p* < 0.05) on the phenolic recovery and its effect was positive. As demonstrated in previous studies, the type of extraction solvent had a significant effect (*p* < 0.05) on the phenolic recovery from PH [5,23,24]. It is also noteworthy that some authors have reported that the phenolic extraction from PH can be divided into two stages: an initial rapid step followed by a step in which the phenolic extraction increases slowly with time [17,25,26]. Therefore, given that the minimal stirring time tested in the present work was 30 min, it may have passed the first step and entered the second step, where the increase in phenolic recovery with the stirring time is minimal. This finding indicates that the stirring time has a minimal impact on the phenolic recovery within the tested range. Furthermore, the lack of fit was not statistically significant (*p* > 0.05) for the calculated model of CSE using water, thereby demonstrating a significant relationship between phenolic recovery and the independent variables. However, for CSE performed with a hydroalcoholic mixture, the lack of fit was statistically significant (*p* < 0.05). This finding suggests the presence of systematic variation that has not been adequately addressed in the calculated model. This phenomenon may be attributable to the precise replicate values of the independent variables in the model, which provide an estimate of pure error [27].

**Figure 1:**
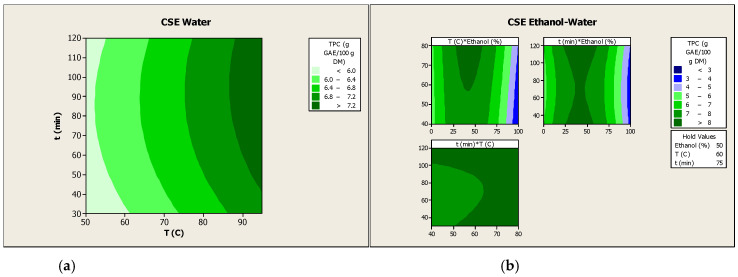
Contour plots between the coupled independent variables of CSE performed with water (**a**) or with ethanol and water (**b**) for the recovery of the phenolic compounds from PH. The symbol “*” represents the interaction between each pair of variables in the contour plots.

Furthermore, the contour plots illustrate the combined effects of each pair of independent variables on the phenolic recovery from PH (Figure 1). However, the corresponding interaction terms were not statistically significant (*p* > 0.05) for the CSE using both types of solvents.

The optimal values of the analyzed independent variables were calculated using the derived equations of both mathematical models in order to obtain the maximum recovery of phenolics from PH. The optimization results indicated that the optimal parameters for the CSE carried out with water, with a solid load of 2% (*w*/*w*), were a temperature of 95 °C and a stirring time of 30 min. It is important to note that, since stirring time was not a significant variable in the recovery of the phenolics from PH, its optimal value was the lowest of the range analyzed by the experimental design. According to the calculations performed, the estimated phenolic recovery was 7.49 g GAE/100 g DM. Regarding the CSE of PH performed with a mixture of ethanol and water, the values of the optimized variables were as follows: a temperature of 80 °C, a stirring time of 30 min and 42% (*v*/*v*) of ethanol in the hydroalcoholic mixture. For this type of extraction, it would be expected that extraction under optimal conditions could involve using the minimum values of the studied range for temperature and stirring time, since both variables were not statistically significant (*p* > 0.05). However, while the calculated optimum value for stirring time aligns with this behavior, in the case of temperature, the calculated optimum value did not correspond to the lowest value within the studied range. Additionally, the contour plot illustrating the relationship between temperature and ethanol concentration demonstrated that higher temperatures, up to 55 °C, resulted in enhanced phenolic recovery. However, the corresponding interaction terms did not reach statistical significance (*p* > 0.05). Under these calculated conditions, the predicted phenolic recovery was 8.82 g GAE/100 g DM. The calculated optimal operation conditions for both extraction solvents were then experimentally validated, and both extraction trials were performed in triplicate. After validation, the phenolic recovery was determined to be 7.06 ± 0.03 g GAE/100 g DM when water was used as the extraction solvent, and 8.16 ± 0.10 g GAE/100 g DM for the extraction performed with a mixture of ethanol and water. These experimental results confirmed that both calculated models were adequate and valid enough to achieve the optimization results. Moreover, these findings indicated that a hydroalcoholic mixture with comparable proportions of ethanol and water led to enhanced phenolic recovery from PH by CSE in comparison to the use of pure solvents (ethanol or water). In this context, it is imperative to acknowledge the polar nature of phenolic compounds. Although these compounds exhibit a greater propensity for hydrophilic interactions compared to lipophilic interaction, their specific hydrophilicity or lipophilicity depends on the number and conjugation of the phenol groups [28]. Notwithstanding the necessity of water for the recovery of phenolic compounds with high polarity, the varying degrees of polarity and solubility of the phenolics contained in PH render hydroalcoholic mixtures more appropriate for the recovery of this type of compounds [8,28]. Furthermore, the results obtained in the present study indicate that within the range of temperatures examined, an increase in extraction temperature leads to an increase in extraction yield. Notably, the highest temperature setting resulted in the greatest extraction yield. This observation is supported by the fact that middle temperatures were used, thus minimizing the possibility of phenolic compounds degradation [28].

The results obtained in the present study exceed those previously documented by other researchers. Therefore, for the CSE of PH using water at 30 °C for 90 min, Tabaraki and Ghadiri [25] reported a value of 4.59 g GAE/100 g DM. For the extraction carried out at 25 °C for 8 h using a solid load of 1/15 (*w*/*v*), Rajaei et al. [17] reported a value of 4.93 g GAE/100 g DM. In addition, Rajaei et al. [29] documented a value of 5.78 g GAE/100 g DM after maintaining the aqueous suspension of PH at 65 °C for 45 min. It should be noted that the aforementioned studies were conducted using the maceration process, without agitation of the extraction media, which could explain the lower yields reported. In addition, the lower temperatures used by these authors may have led to a reduction in phenolic recovery, as temperature was identified as a statistically significant variable in the present study. However, other researchers have reported higher phenolic recovery from PH than that achieved in this study when using maceration with water. Therefore, Seifzadeh et al. [30] attained a recovery of 12.03 g GAE/100 g DM by maintaining PH in water for 8 h at room temperature. Taghizadeh et al. [24] achieved a phenolic recovery of 14.88 g GAE/100 g DM after 48 h of maintaining PH in water at room temperature. Moreover, Azhdari et al. [6] reported a value of phenolic extraction of 9.93 ± 0.25 g GAE/100 g DM from PH following a water treatment (25 °C, 8 h and 1/15 (*w*/*v*) solid load). In regard to the CSE of PH performed with the application of magnetic stirring, Pakdaman et al. [2] achieved a lower phenolic recovery (3.5 g GAE/100 g DM) than that achieved in the present study. The authors reported using water as the extraction solvent and performing the extraction at 65 °C for 2 h. It is important to note that the varying phenolic recovery values resulting from the application of different extraction conditions, as well as the significant differences in the phenolic content of PH among different pistachio genotypes and climatic conditions, affect the extraction yield [31].

Regarding the application of the RSM approach to optimize the operating conditions for the CSE of phenolics from PH, a limited number of studies can be found in the literature. Therefore, Rajaei et al. [17] attained a phenolic recovery of 5.78 ± 0.02 g GAE/100 g DM (maceration with water, at 65 °C for 45 min) and Tabaraki and Ghadiri [25] achieved a value of 4.59 g GAE/100 g DM (water, with magnetic stirrer, at 30 °C for 90 min).

### 3.2. Optimization of Accelerated Solvent Extraction Conditions and Verification of the Models

As was the case with the CSE, the operation variables of the ASE were optimized with the objective of maximizing the phenolic recovery from PH. Therefore, two CCRDs were designed. One comprised two independent variables (time and temperature) for the ASE conducted with water, and the other comprised three independent variables (time, temperature and alcohol percentage) to optimize the extraction with a mixture of ethanol and water. The following tables present the combinations of the ASE variables from PH and the experimental results of the phenolic recovery (g GAE/100 g DM) for the extractions carried out with water and ethanol and water mixtures. Table 3 shows the results for water, while Table 4 presents the results for the ethanol and water mixture. Therefore, the phenolic recovery exhibited a range from 5.40 to 8.85 g GAE/100 g DM (ASE with water) and from 2.98 to 9.52 g GAE/100 g DM (ASE with a mixture of ethanol and water).

Subsequently, two ANOVAs were calculated, one for each experimental design corresponding to each extraction solvent. The objective of the present study was to analyze the obtained results and to assess the effects of the independent variables and their possible interactions in each ASE trial, depending on the type of extraction solvent used. The calculated quadratic models explained 99.19% of the variation in the phenolic recovery from PH by ASE using water (Table S5) and 91.06% of the variation in the phenolic recovery using the ethanol and water mixture (Table S6). Consequently, the calculated models for the ASE performed with both extraction solvents exhibited a satisfactory degree of correlation with the experimental data. Moreover, the mathematical models demonstrated statistical significance at the 95% confidence level, with *p*-values less than 0.05 (0.000 for the two calculated models) (Tables S7 and S8).

In the case of the ASE performed with water, the extraction time exhibited a greater influence on the phenolic recovery from PH than the temperature. Both variables demonstrated a significant (*p* < 0.05) and positive effect. Conversely, the ASE conducted with an ethanol and water mixture revealed that none of the independent variables, linear or quadratic, or their interactions, attained statistical significance for the mathematical model (*p* > 0.05). However, it is noteworthy that temperature exerted a positive and higher influence compared to ethanol percentage and extraction time. It is plausible that this phenomenon is attributable to polarity changes resulting from an increase in temperature. Despite its higher polarity compared to ethanol, the elevated pressure and temperature that exceed its boiling point result in a reduction in water polarity due to the dissociation of intermolecular hydrogen bonds [31]. This solvent polarity change, induced by an increase in temperature, may exert a more substantial influence on phenolic extraction than the ethanol percentage and the extraction time.

**Figure 2:**
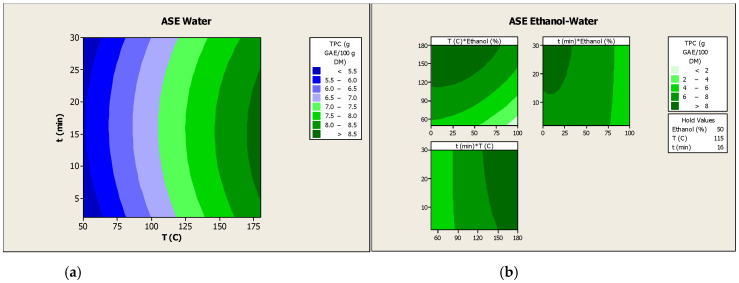
Contour plots between the coupled independent variables of ASE performed with water (**a**) or with ethanol and water (**b**) for the recovery of the phenolic compounds from PH. The symbol “*” represents the interaction between each pair of variables in the contour plots.

Furthermore, while for the calculated model of water-based ASE showed no statistically significant lack of fit (*p* > 0.05), the lack of fit for the ASE performed with a hydroalcoholic mixture was statistically significant (*p* < 0.05). This fact demonstrates a significant relationship between phenolic recovery and the independent variables of the water-based ASE model. In contrast, the calculated model for the ASE performed with a hydroalcoholic mixture exhibited a statistically significant lack of fit. This suggests the presence of systematic variation that was not accounted for by the model. This could be attributed to the replicated values of the independent variables in the model [27]. In addition, the contour plots illustrate the combined effects of each pair of independent variables on the phenolic recovery from PH (Figure 2). Nevertheless, the corresponding interaction terms did not attain statistical significance (*p* > 0.05) when both type of solvents were used.

The optimal values of the evaluated independent variables were calculated according to the equations of the mathematical model corresponding to each extraction solvent. This calculation was performed with the objective of maximizing the phenolic recovery from PH. The optimization results indicated that the optimal parameters for the ASE of PH performed with water were a temperature of 180 °C and an extraction time of 15 min. In the context of these optimized conditions, the estimated phenolic recovery was determined to be 8.69 g GAE/100 g DM. With regard to the ASE of PH conducted with an ethanol and water mixture, the optimal values for the variables were determined to be 180 °C for 15 min and 23% (*v*/*v*) of ethanol. According to the model, while temperature demonstrated no significant effect (*p* > 0.05), the contour plot between temperature and ethanol percentage exhibited higher phenolic recovery at elevated temperatures. In addition, given the established negative correlation between time and ethanol concentration on phenolic recovery, it was hypothesized that values of 2 min and 0% of ethanol, representing the minimum levels of both variables in the experimental design, would yield optimal phenolic recovery. However, the contour plot depicting the relationship between extraction time and ethanol concentration revealed that higher values yielded greater phenolic recovery. With regard to the ethanol concentration, the use of water as a solvent for extraction (0% ethanol) was analyzed in the corresponding optimization. Therefore, under the aforementioned calculated optimal conditions, the predicted phenolic recovery from PH by ASE using an ethanol and water mixture was 9.65 g GAE/100 g DM. This result indicates a higher phenolic recovery than that expected when ASE was carried out only with water (8.69 g GAE/100 g DM). The experimental validation of both models was conducted through the process of PH extraction by ASE under conditions that were calculated to be optimal for operation. These optimal extraction trials were performed in triplicate.

Subsequent to the validation process, the achieved values of phenolic recovery were ascertained to be 8.98 ± 0.11 g GAE/100 g DM (water) and 9.92 ± 0.09 g GAE/100 g DM (ethanol and water mixture). Despite minor discrepancies between the calculated and experimental values, the findings substantiated the efficacy and dependability of the response models in estimating the optimization outcomes.

Notably, the phenolic recovery from PH was higher using ASE than CSE under optimal conditions. This phenomenon can be attributed to enhanced extraction kinetics and yields resulting from temperatures above the solvent boiling point and elevated pressures [8,10]. Moreover, the highest yield of phenolic recovery from PH was observed by ASE when an ethanol and water mixture was used as the extraction solvent. In order to elucidate this phenomenon, it is imperative to acknowledge the inherent properties of phenolic compounds, which are predominantly polar and manifest a heightened degree of hydrophilicity in comparison to lipophilicity. Consequently, polar protic solvents have been shown to generally yield superior extraction results [28]. The polarity of water is known to decrease due to elevated pressure and temperature, and the use of ethanol as a co-solvent has been shown to reduce the thermal hydrolysis and degradation [31]. These factors contribute to an increase in phenolic recovery. The properties of the ethanol and water mixtures render them well suited for high-pressure and high-temperature techniques, such as ASE [8,31].

To the best of our knowledge, this is the first instance in which ASE has been applied to PH to valorize this specific agri-food waste. To date, no studies have been identified in the literature that focus on the recovery of high-value compounds from any type of pistachio by-product using ASE. In the context of alternative extraction techniques applied to PH, previous studies have reported lower phenolic recovery values than those obtained in the present study. Accordingly, Rajaei et al. [17] have documented the phenolic recovery of 6.095 ± 0.069 and 6.102 ± 0.143 g GAE/100 g DM from PH through UAE and MAE, respectively. It should be noted that both techniques were performed using water under optimized conditions. Consequently, the researchers determined that the optimal conditions for UAE and MAE, were as follows: a solid load of 20% (*v*/*w*), at 65 °C for 25 min and a solid load of 20% (*v*/*w*), at 65 °C for 45 min, respectively. Similarly, Tabaraki and Ghadiri [25] reported phenolic recoveries of 5.81 and 6.26 g GAE/100 g DM from PH, through UAE and MAE, respectively, using water as the extraction solvent, under optimized conditions (UAE: solvent to solid ratio of 50:1, at 30 °C for 45 min; MAE: solvent to ratio of 100:3, 300 W, 150 s of irradiance time and total time of 21 min). In addition, Özbek et al. [5] obtained a phenolic recovery of 6.22 g GAE/100 g DM from PH by MAE using a mixture of ethanol and water (56% ethanol), a solvent to solid ratio of 19:1, and applying a microwave power of 140 W for 4.5 min. In regard to other pistachio by-products, Zalazar-García et al. [12] obtained a phenolic yield of 7.3 g GAE/100 g DM from pistachio industry waste using an ethanol and water mixture under UAE optimized conditions (45.5% *v*/*v* ethanol and 0.75 h of extraction time). However, the lower extraction yields achieved by those authors may be attributable to the use of different extraction techniques than that used in the present study. Moreover, the use of lower temperatures and higher solvent to solid ratios could potentially reduce the phenolic recovery yield. In addition, it can be concluded that, while phenolic extraction yield is contingent on cultivar type, soil condition, climate, geographical location and processing methods [1], the ASE technique is highly adequate for PH valorization. This assertion is supported by the superior phenolic recovery results obtained in the present study in comparison to those reported in the literature through the use of other green extraction techniques.

Furthermore, the extraction from PH performed with ASE (23% ethanol, 180 °C, 15 min) achieved 22% more phenolic recovery than that carried out by CSE (42% ethanol, 80 °C, 30 min). Consequently, although ASE requires higher pressure and temperature than CSE, the reduced solvent volume, shorter extraction time and higher thermal efficiency suggest that energy consumption may be similar for the two extraction techniques. Moreover, the percentage of ethanol used in the hydroalcoholic mixture under optimal conditions of ASE is lower than that used by CSE under optimal conditions. Therefore, within the context of the operation costs, the utilization of ASE could prove advantageous. Furthermore, while the high cost of the equipment could hinder its implementation on an industrial scale, the notable capabilities of ASE can effectively balance out the initial investment in a relatively brief timeframe [8]. However, further research is necessary to determine the feasibility and economic viability of scaling up the process.

### 3.3. Composition and Antioxidant Activities of the Optimal Extracts of Pistachio Hull

The extracts obtained from PH under optimal conditions were subjected to analysis in order to quantify not only TPC, but also TFC, TTC, TPrC and THTC. In addition, twelve individual phenolic compounds were determined by HPLC-DAD, and the antioxidant activities were measured using two assays: FRAP and DPPH radical-scavenging activity (RSA).

#### 3.3.1. Chemical Composition

As shown in Table 5, the highest results of TPC, TFC, TTC, TPrC and THTC were achieved through the application of ASE with an ethanol and water mixture (9.92 ± 0.09 g GAE/100 g DM; 1.98 ± 0.14 g CE/100 g DM; 6.63 ± 0.07 g GAE/100 g DM; 5.98 ± 0.11 g GAE/100 g DM and 0.64 ± 0.04 g GAE/100 g DM, respectively) (*p* < 0.05). In the case of CSE from PH, the use of an ethanol and water mixture as extraction solvent resulted in a higher yield of TPC, TTC and THTC than with the use of water (*p* < 0.05). In contrast, the use of water or a mixture of ethanol and water for the extraction from PH by CSE yielded equivalent recoveries of TFC and TPrC (*p* < 0.05). The findings indicate that while ethanol and water mixtures are more effective for extracting a wider range of phenolic compounds, including tannins, in the case of the extraction of flavonoids and proanthocyanidins by CSE, water alone is sufficient for their recovery, probably due to their high relatively polarity and good solubility in water. Additionally, these compounds can be efficiently extracted with water alone and the addition of ethanol does not significantly enhance their recovery by CSE. Moreover, the elevated temperature employed in the CSE with water (95 °C) has the potential to enhance solubility and diffusion rates. However, when extraction is carried out by ASE, the use of high pressures and temperatures makes it more favorable to use hydroalcoholic mixtures to increase not only the phenolic recovery (see Section 3.2) but also each phenolic fraction recovery.

The observed concentrations of the flavonoids recovered from PH were found to be slightly lower than the concentrations reported by Azhdari et al. [6]. Therefore, these researchers documented the recovery of flavonoids in PH extracts prepared by CSE using an ethanol and water mixture with a solvent to solid ratio of 15:1 at 25 °C for 8 h, yielding a recovery of 2.80 ± 0.01 g CE/100 g DM. However, the recovery of phenolic compounds attained by Azhdari et al. [6] was similar to that achieved in the present study. In addition, Taghizadeh et al. [24] achieved a flavonoid recovery in PH extracts prepared by CSE using pure water or pure ethanol, with a solvent to solid ratio of 10:1, at room temperature for 48 h, that were 10-fold higher than those attained in the present study. The observed differences can be ascribed to the utilization of disparate pistachio varieties, geographical location and climatic conditions [32]. In contrast, the findings on total tannin recovery were in agreement with those reported by Arjeh et al. [1] (3.75 g GAE/100 g DM with water and 5.37 g GAE/100 g DM using ethanol). However, the aforementioned authors documented higher flavonoid recovery from the extraction of PH with water (4.2 g CE/100 g DM) and with ethanol (2.9 g CE/100 g DM) when compared with the results of the present study. In the context of other pistachio by-product extracts, the findings of this study demonstrated that the recovered flavonoid content was higher than that reported by Zalazar-García et al. [12]. The latter researchers obtained a value of 0.34 g CE/100 g DM in pistachio industry waste extracts through UAE using an hydroalcoholic mixture with 45.5% ethanol for 4.5 h. However, it is important to note that the by-product in question consists not only of hulls but also of twigs, stems and leaves.

**Figure 3:**
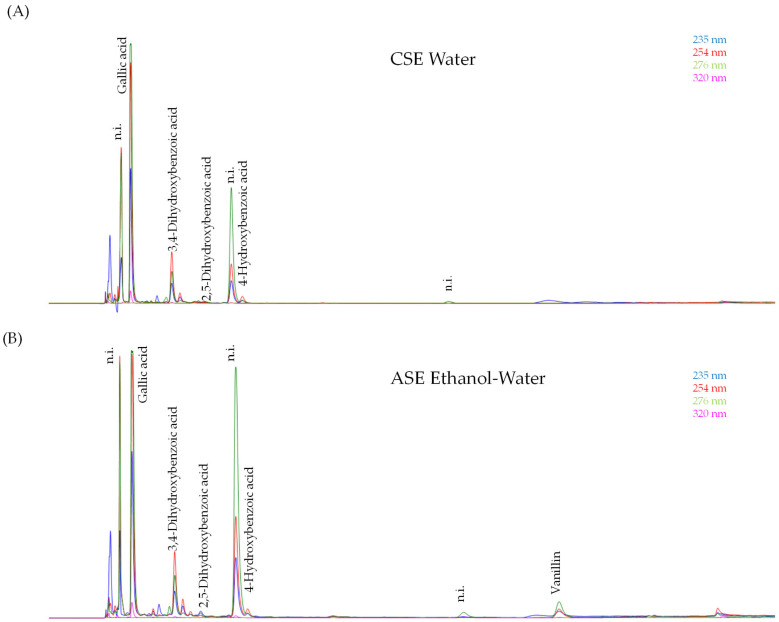
HPLC-DAD chromatograms of the PH optimal extracts obtained by water-based CSE (**A**) and by ethanol-water ASE (**B**). n.i.: Not identified.

With respect to the individual phenolic compounds analyzed in the optimal PH extracts, the results indicated that their content depends on the type of extraction (Table 5). Figure 3 shows the chromatographic separation of the optimal PH extract obtained by CSE using water, as well as the optimal hydroalcoholic extract generated by ASE. The predominant compound in all of the optimal extracts was gallic acid, which achieved the highest recovery when PH was subjected to ASE with an ethanol and water mixture or with water (33.86 ± 0.34 and 33.37 ± 1.79 mg/g DM, respectively) (*p* < 0.05). The presence of gallic acid as the predominate phenolic compound in PH extracts has been documented in several studies [1,23,26,33]. Therefore, the following values of gallic acid recovery from PH have been previously reported: 0.005–0.006 mg/g DM [34], 0.015–0.057 mg/g DM [35], 3.07 mg/g DM [23] and 6.31–22.2 mg/g DM [33]. This variability can be attributed to the fact that this compound can be present in PH as simple gallic acid or as highly complex gallotannins [1]. In this study, prior to quantifying the individual phenolics present in the optimal extract samples, acid hydrolysis with HCl (final concentration 2.5 M) for 1 h at 90 °C [8] was carried out. This hydrolysis treatment has enhanced the heat-dependent degradation of the gallotannins, which may result in an observed increase in gallic acid levels [8,33]. Therefore, the gallic acid contained in the initial optimal extracts has been quantified, as well as a significant amount of gallic acid liberated from gallotannins during the hydrolysis treatment prior to analysis. Consequently, it is noteworthy that a significant proportion of the phenolics was identified as gallic acid (35.7–48.1%). However, these percentages are lower than those previously reported by Ersan et al. [33], who observed that gallic acid constituted 74–92% of the total phenolics contained in the PH extracts obtained through subcritical water extraction at a pressure of 6.9 MPa in the range of 170 and 190 °C and a flow rate of 4 mL/min. Therefore, the findings of Ersan et al. [33] indicate that the majority of the gallic acid present in the gallotannins of the PH has undergone hydrolysis.

Furthermore, the second most abundant compound was 3,4-dihydroxybenzoic acid, with maximum values observed in extracts generated by ASE using water or a mixture of ethanol and water (4.80 ± 0.24 and 4.59 ± 0.06 mg/g DM, respectively) (*p* < 0.05). With regard to other individual phenolic compounds, 4-hydroxybenzoic acid merits particular attention, as it exhibited the highest recovery in the extracts obtained by ASE using water (0.68 ± 0.02 mg/g DM). In a similar manner, the recovery of 2,5-dihydroxybenzoic acid was highest in extracts generated by ASE (0.36 ± 0.02 mg/g DM for both extraction solvents). Moreover, vanillin was exclusively detected in extracts generated through the use of an ethanol and water mixture and the highest recovery was achieved by CSE (6.00 ± 0.17 mg/g DM). In addition, 3-hydroxybenzoic acid, vanillic acid, caffeic acid, syringic acid, *p*-coumaric acid, syringaldehyde and ferulic acid were not detected in any of the optimal PH extracts. These results are in agreement with those previously documented by other researchers, who following the highest content of gallic acid, they have identified 3,4-dihydroxybenzoic acid and 4-hydroxybenzoic acid as the most abundant phenolic acids in PH extracts [1,23,35]. Furthermore, the quantities of 4-hydroxybenzoic acid recovered by the aforementioned researchers (0.004–2.06 mg/g DM) were comparable to those obtained in the present study, while the amounts of 3,4-dihydroxybenzoic acid were lower (0.004–0.32 mg/g DM). Moreover, previous studies have documented the presence of vanillic, caffeic, syringic, ferulic and *p*-coumaric acids in PH extracts [1,23,26]. However, these compounds were not detected in the optimal extracts obtained in the present study. In addition, other authors have reported the presence of other phenolic compounds in PH extracts, including catechin, epicatechin, quercetin, kaempferol, luteolin, apigenin and naringenin [1,23,34,35]. Nevertheless, the chromatographic method used in the present study for the analysis of the individual phenolic compounds was unable to determine the aforementioned compounds.

#### 3.3.2. Antioxidant Activities

Regarding the antioxidant properties of the optimal PH extracts, the highest FRAP values were obtained from the ASE, with no statistically significant differences (*p* < 0.05) observed between the use of the two extraction solvents (7.23 ± 0.06 g AAE/100 g DM with water and 7.18 ± 0.07 g AAE/100 g with the ethanol and water mixture). Therefore, it can be concluded that an increase in the phenolic content of the PH extracts leads to an increase in their antioxidant activity. These results are in agreement with those reported by other authors in previous studies which also conclude that there is a lineal and positive correlation between the concentration of phenolic compounds and their antioxidant activity due to their ability to donate hydrogen [16,26,36].

**Figure 4:**
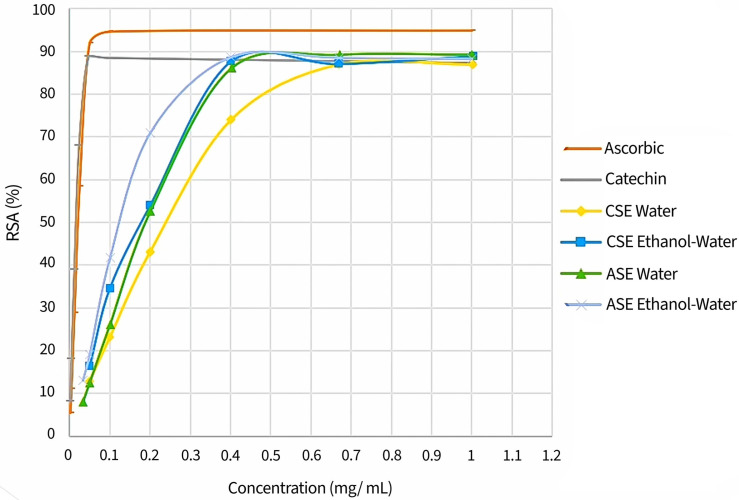
DPPH free radical-scavenging activity (RSA) of PH extracts concentrations obtained by CSE and ASE under optimal conditions and antioxidant reference compounds (ascorbic acid and catechin).

For the DPPH radical-scavenging assay, RSA values were expressed as a percentage of the decrease in the absorbance of the sample compared to the absorbance of the DPPH solution without the PH extract at 517 nm [8]. The scavenging activity of the optimal extracts on DPPH radicals exhibited a concentration-dependent increase, reaching values that were comparable to those achieved by reference antioxidant compounds, such as ascorbic acid and catechin (Figure 4). These findings are in agreement with those of previous studies, which reported similar or higher antioxidant properties in PH extracts prepared by maceration with water at room temperature overnight, when compared to synthetic antioxidants such as trolox and butylhydroxytoluene (BHT) [29,32]. The observed benefit can be attributed to the high concentration of gallic acid in PH extracts. Previous research has observed that gallic acid possesses strong antioxidant properties, which have been shown to confer antidiabetic, antimicrobial and antimutagenic properties to the extract [1,32]. Furthermore, the additive and synergistic effects of the complex mixture of phytochemicals present in PH extracts may be responsible for their robust antioxidant properties.

As shown in Table 5, the highest RSA_max_ values (*p* < 0.05) were observed in the PH extracts obtained by ASE using the ethanol and water mixture or pure water and by CSE using the ethanol and water mixture (90.0 ± 0.3% at 0.45 ± 0.00 mg/mL; 89.7 ± 0.0% at 0.50 ± 0.00 mg/mL and 89.9 ± 0.2% at 0.48 ± 0.00 mg/mL, respectively). Similarly, extracts prepared by ASE using a combination of ethanol and water exhibited the most favorable IC50 values (*p* < 0.05) (0.16 ± 0.00 mg/mL). It is noteworthy that the IC50 values of antioxidant compounds like ascorbic acid and catechin, determined under identical conditions, were found to be 0.02 mg/mL for both compounds. Therefore, an extract prepared from an amount of PH that is eight times higher than those solutions prepared from ascorbic acid or catechin achieved similar antioxidant properties. The results of this study suggest a potential approach for the utilization of PH as an antioxidant additive.

The results of the FRAP assay and RSA_max_ attained in the present study were higher than those observed by Tabaraki and Ghadari [25]. These researchers studied the production of PH extracts with water through CSE (solvent to solid ratio 50:1, particle size of 0.25 mm, 30 °C, for 90 min, with magnetic stirrer), UAE (solvent to solid ratio 50:1, particle size of 0.25 mm, 30 °C, for 45 min) and MAE (solvent to ratio 100:3, 300 W, 150 s of irradiance time and total time of 21 min). They obtained values for FRAP of 3.35, 3.53 and 4.48 g AAE/100 g DM, respectively. Similarly, the aforementioned authors observed values for RSA_max_ of 80.5, 84.2 and 74.6%, respectively. In addition, the IC50 values obtained in the present study were lower than those documented by Özbek et al. [23] for the PH extract generated using a hydroalcoholic mixture (40% ethanol) through MAE (solvent to solid ratio 19:1, 140 W for 4.5 min) (0.70 ± 0.04 mg/mL).

It is therefore important to note that natural extracts obtained from PH in a concentration 10-fold higher than solutions of synthetic compounds (ascorbic acid or catechin) can achieve comparable values of RSA_max_. This could be a viable alternative to valorize this by-product, thus avoiding the environmental problems currently caused by PH. In addition, there are several biological benefits associated with the consumption of bioactive compounds from vegetable sources [1]. Moreover, the implementation of eco-friendly extraction methods, such as ASE, along with the use of safe recyclable solvents, enhances the sustainability of the process.

## 4. Conclusions

The results of this study demonstrate for the first time that ASE was effective in enhancing the extraction yield of phenolic compounds from PH using eco-friendly solvents. The optimization of ASE and CSE conditions for the extraction of phenolic compounds from PH using RSM with CCRD has proved to be an effective approach for experimental design and process optimization. The optimal conditions that were calculated through mathematical models included 23% ethanol in water at 180 °C for 15 min for ASE and 42% ethanol in water at 80 °C for 30 min for CSE. Under optimized conditions, experimental values exhibited strong agreement with the values predicted by the models. Furthermore, the ASE process proved to be more efficacious than the CSE process in the context of phenolic extraction from PH. This was evidenced by a phenolic recovery value of 9.92 ± 0.09 g GAE/100 g DM, which was observed to be significantly higher than the maximum value of 8.16 ± 0.10 g GAE/100 g DM attained through the CSE method. Consequently, in addition to the higher phenolic recovery value obtained by ASE, the use of lower percentages of ethanol in the hydroalcoholic mixture and shorter times renders ASE a promising alternative for the recovery of bioactive compounds from PH. Furthermore, of the twelve phenolic compounds that were analyzed in the optimal extracts of PH, gallic acid was identified as the predominant compound (35.7–48.1% of the TPC), followed by 3,4-dihydroxybenzoic acid, 4-hydroxybenzoic acid and 2,5-dihydroxybenzoic acid. Vanillin was only detected in extracts generated using an ethanol and water mixture. In contrast, 3-hydroxybenzoic acid, vanillic acid, caffeic acid, syringic acid, *p*-coumaric acid, syringaldehyde and ferulic acid were not identified.

The results of this study indicated that PH is a good source of phenolic compounds with high antioxidant properties. Additionally, ASE has emerged as a promising and environmentally friendly method for the recovery of bioactive compounds from agri-food processing by-products, such as PH. These findings could enhance utilization of PH extracts in food, animal feed, cosmetic and nutraceutical industries, reducing waste and promoting the bioeconomy development. However, further studies should be developed to assess the scalability and economic feasibility of the process. Moreover, research should be conducted to determine the bioactive roles of the active compounds from PH in industrial products. This would contribute to more effective promotion of the use of agri-food by-products.

## Figures and Tables

**Table 1:** Experimental design of the CSE of PH with water and the observed results of the total phenolic content (TPC) recovered (g GAE/100 g DM).

Trial	T (°C)	t (min)	TPC (g GAE/100 g DM)
1	57	107	6.01
2	57	43	5.76
3	88	43	6.98
4	88	107	7.34
5	73	120	6.61
6	73	30	6.50
7	73	75	6.33
8	73	75	6.77
9	73	75	6.81
10	73	75	6.64
11	73	75	6.84
12	95	75	7.38
13	50	75	6.10

**Table 2:** Experimental design of the CSE of PH with ethanol and water and the observed results of the total phenolic content (TPC) recovered (g GAE/100 g DM).

Trial	Ethanol (%, *v*/*v*)	T (°C)	t (min)	TPC (g GAE/100 g DM)
1	80	48	48	6.23
2	80	48	102	6.59
3	80	72	102	7.11
4	80	72	48	6.96
5	50	40	75	7.46
6	50	60	75	7.86
7	50	60	75	7.97
8	50	60	75	8.05
9	50	60	75	8.01
10	50	60	75	8.01
11	50	60	75	7.89
12	50	60	30	7.98
13	50	60	120	7.94
14	50	80	75	7.72
15	0	60	75	5.99
16	100	60	75	2.28
17	20	48	48	7.19
18	20	48	102	7.55
19	20	72	102	7.72
20	20	72	48	7.72

**Table 3:** Experimental design of the ASE of PH with water and the observed results of the total phenolic content (TPC) recovered (g GAE/100 g DM).

Trial	T (°C)	t (min)	TPC (g GAE/100 g DM)
1	69	6	5.79
2	69	26	5.85
3	115	30	6.92
4	115	2	6.98
5	115	16	7.26
6	115	16	7.19
7	115	16	7.34
8	115	16	7.25
9	115	16	7.18
10	50	16	5.40
11	180	16	8.85
12	161	6	8.00
13	161	26	7.97

**Table 4:** Experimental designs of the ASE of PH with ethanol and water and the observed results of the total phenolic content (TPC) recovered (g GAE/100 g DM).

Trial	Ethanol (%, *v*/*v*)	T (°C)	t (min)	TPC (g GAE/100 g DM)
1	80	76	8	4.65
2	80	76	24	4.49
3	80	154	8	6.97
4	80	154	24	7.30
5	50	50	16	2.98
6	50	115	16	7.01
7	50	115	16	7.49
8	50	115	16	7.29
9	50	115	16	7.48
10	50	115	16	7.48
11	50	115	16	7.58
12	50	115	2	6.72
13	50	115	30	7.32
14	50	180	16	9.52
15	0	115	16	7.48
16	100	115	16	4.67
17	20	76	8	7.20
18	20	76	24	7.29
19	20	154	24	9.00
20	20	154	8	8.62

**Table 5:** Chemical composition and antioxidant activities (2.2-diphenyl-1-picrylhydrazyl (DPPH) free radical-scavenging activity and ferric reducing antioxidant power (FRAP)) of PH extracts obtained by CSE or ASE using water or a mixture of ethanol and water under optimal conditions. Different letters indicate significant differences (Tukey’s HDS; *p* < 0.05) between PH extracts for the same parameter.

	CSE Water	CSE Ethanol/Water	ASE Water	ASE Ethanol/Water
Optimal conditions	95 °C, 30 min	42% Ethanol, 80 °C, 30 min	180 °C, 15 min	23% Ethanol, 180 °C, 15 min
**Chemical composition**				
TPC (g GAE/100 g DM)	7.06 ± 0.03 _D_	8.16 ± 0.10 _C_	8.98 ± 0.11 _B_	9.92 ± 0.09 _A_
TFC (g CE/100 g DM)	1.34 ± 0.08 _B_	1.49 ± 0.03 _B_	1.46 ± 0.09 _B_	1.98 ± 0.14 _A_
TTC (g GAE/100 g DM)	4.67 ± 0.10 _D_	5.02 ± 0.08 _C_	6.09 ± 0.20 _B_	6.63 ± 0.07 _A_
TPrC (g GAE/100 g DM)	4.58 ± 0.07 _C_	4.62 ± 0.09 _C_	5.63 ± 0.17 _B_	5.98 ± 0.11 _A_
THTC (g GAE/100 g DM)	0.09 ± 0.03 _C_	0.40 ± 0.02 _B_	0.46 ± 0.07 _B_	0.64 ± 0.04 _A_
Gallic acid (mg/g DM)	28.70 ± 0.35 _B_	29.15 ± 0.49 _B_	33.37 ± 1.79 _A_	33.86 ± 0.34 _A_
3,4-Dihydroxybenzoic acid (mg/g DM)	3.83 ± 0.02 _B_	3.60 ± 0.12 _C_	4.80 ± 0.24 _A_	4.59 ± 0.06 _A_
2,5-Dihydroxybenzoic acid (mg/g DM)	0.14 ± 0.00 _C_	0.25 ± 0.00 _B_	0.36 ± 0.02 _A_	0.36 ± 0.02 _A_
4-Hydroxybenzoic acid (mg/g DM)	0.49 ± 0.00 _C_	0.39 ± 0.00 _D_	0.68 ± 0.02 _A_	0.56 ± 0.01 _B_
3-Hydroxybenzoic acid (mg/g DM)	nd	nd	nd	nd
Vanillic acid (mg/g DM)	nd	nd	nd	nd
Caffeic acid (mg/g DM)	nd	nd	nd	nd
Syringic acid (mg/g DM)	nd	nd	nd	nd
Vanillin (mg/g DM)	nd	6.00 ± 0.17 _A_	nd	3.52 ± 0.72 _B_
*p*-Coumaric acid (mg/g DM)	nd	nd	nd	nd
Syringaldehyde (mg/g DM)	nd	nd	nd	nd
Ferulic acid (mg/g DM)	nd	nd	nd	nd
**Ferric reducing antioxidant power (FRAP)**				
FRAP (g AAE/100 g DM)	6.65 ± 0.07 _C_	6.85 ± 0.07 _B_	7.23 ± 0.06 _A_	7.18 ± 0.07 _A_
**DPPH free radical-scavenging activity**				
RSA_max_ (%)	87.3 ± 0.1 _B_	89.9 ± 0.2 _A_	89.7 ± 0.0 _A_	90.0 ± 0.3 _A_
C RSA_max_ (mg/mL)	0.67 ± 0.00 _A_	0.48 ± 0.00 _C_	0.50 ± 0.00 _B_	0.45 ± 0.00 _D_
IC50 (mg/mL)	0.26 ± 0.00 _A_	0.20 ± 0.00 _B_	0.19 ± 0.00 _C_	0.16 ± 0.00 _D_

TPC: Total phenolic content; TFC: Total flavonoid content; TTC: Total tannin content; TPrC: Total Proanthocyanidin content; THTC: Total hydrolyzable tannin content; RSA_max_: Maximum DPPH radical-scavenging activity; C RSA_max_: Concentration of a substance that provides maximum DPPH radical-scavenging activity; IC50: Concentration of a substance that provides 50% of DPPH radical-scavenging activity; nd: Not detected; GAE: Gallic acid equivalents; CE: Catechin equivalents; AAE: Ascorbic acid equivalents; Ascorbic acid (RSA_max_: 94.7 ± 0.2%; C RSA_max_: 0.05 ± 0.00 mg/mL; IC50: 0.02 ± 0.00 mg/mL); Catechin (RSA_max_: 88.8 ± 0.2%; C RSA_max_: 0.05 ± 0.00 mg/mL; IC50: 0.02 ± 0.00 mg/mL).

## Data Availability

The original contributions presented in this study are included in the article and Supplementary Materials. Further inquiries can be directed to the corresponding authors.

## Supplementary Materials

The following supporting information can be downloaded at: https://www.mdpi.com/article/10.3390/antiox15050558/s1, Table S1: Estimated regression coefficients for the recovery of phenolic compounds from PH by CSE using water. Note: This model includes all the terms; Table S2: Estimated regression coefficients for the recovery of phenolic compounds from PH by CSE using water–ethanol. Note: This model includes all the terms; Table S3: Analysis of variance for the recovery of phenolic compounds from PH by CSE using water. Note: This model includes all the terms; Table S4: Analysis of variance for the recovery of phenolic compounds from PH by CSE using water–ethanol. Note: This model includes all the terms; Table S5: Estimated regression coefficients for the recovery of phenolic compounds from PH by ASE using water. Note: This model includes all the terms; Table S6: Estimated regression coefficients for the recovery of phenolic compounds from PH by ASE using water–ethanol. Note: This model includes all the terms; Table S7: Analysis of variance for the recovery of phenolic compounds from PH by ASE using water. Note: This model includes all the terms; Table S8: Analysis of variance for the recovery of phenolic compounds from PH by ASE using water–ethanol. Note: This model includes all the terms.

## Abbreviations

The following abbreviations are used in this manuscript:

AAE	Ascorbic acid equivalents
ANOVA	Analysis of variance
ASE	Accelerated solvent extraction
CCRD	Central composite rotatable design
CE	Catechin equivalents
CRSA_max_	Concentration of the extract that provides the maximum radical-scavenging activity
CSE	Conventional solvent extraction
DAD	Diode array detector
DM	Dry matter
DPPH	α,α-diphenyl-β-picrylhydrazyl
FRAP	Ferric reducing antioxidant power
GAE	Gallic acid equivalents
GRAS	Generally recognized as safe
HPLC	High-performance liquid chromatography
IC50	Concentration of the extract that provides 50% of the radical-scavenging activity
MAE	Microwave-assisted extraction
PH	Pistachio hull
RSA	Radical-scavenging activity
RSA_max_	Maximum radical-scavenging activity
RSM	Response surface methodology
TFC	Total flavonoid content
THTC	Total hydrolyzable tannin content
TPC	Total phenolic content
TPrC	Total proanthocyanidin content
TTC	Total tannin content
UAE	Ultrasonic-assisted extraction
