# Supplementary material for: Optimization of Phenolic Compounds Recovery from Pistachio Hull Using Accelerated Solvent Extraction
Source: Antioxidants (Basel). 2026 Apr 28;15(5):558. doi: 10.3390/antiox15050558 (PMC13203263; doi:10.3390/antiox15050558)
Supplement: Supplementary file 1 [file antioxidants-15-00558-s001.zip › antioxidants-4225379-supplementary.pdf]

Supplementary material

Table S1. Estimated regression coefficients for the recovery of phenolic compounds from PH by CSE using water. Note: This model includes all the terms. The symbol "*" represents the interaction between variables.

Term	Coefficient	Standard error coef.	T	p
Constant	6.67749	0.09266	72.062	0.000
T (°C)	0.54289	0.07326	7.411	0.000
t (h)	0.09593	0.07326	1.310	0.232
T (°C) * T (°C)	0.00022	0.07856	0.003	0.998
t (h) * t (h)	-0.09290	0.07856	-1.183	0.276
T (°C) * t (h)	0.02592	0.10360	0.250	0.810
S = 0.207200	PRESS=1.18062			
R-square = 89.25%	R-square (pred) = 57.77%	R-square (adj) = 81.57%		

Table S2. Estimated regression coefficients for the recovery of phenolic compounds from PH by CSE using water–ethanol. Note: This model includes all the terms. The symbol "*" represents the interaction between variables.

Term	Coefficient	Standard error coef.	T	p
Constant	5.53139	5.23348	1.057	0.315
Ethanol (%)	0.10072	0.04185	2.406	0.037
T (°C)	0.01653	0.13588	0.122	0.906
t (h)	-0.01435	0.04963	-0.289	0.778
Ethanol (%) * Ethanol (%)	-0.00138	0.00017	-8.304	0.000
T (°C) * T (°C)	0.00004	0.00103	0.042	0.968
t (h) * t (h)	0.00019	0.00020	0.944	0.367
Ethanol (%) * T (°C)	0.00019	0.00056	0.350	0.733
Ethanol (%) * t (h)	0.00002	0.00025	0.094	0.927
T (°C) * t (h)	-0.00022	0.00061	-0.367	0.721
S = 0.117661	PRESS=0.555983			
R-square = 90.55%	R-square (pred) = 28.70%	R-square (adj) = 82.04%		

Table S3. Analysis of variance for the recovery of phenolic compounds from PH by CSE using water. Note: This model includes all the terms. The symbol "*" represents the interaction between variables.

Source	df	Sum of squares Seq.	Sum of squares Adjust.	Mean squares Adjust.	F	p
Regression	5	2.4953	2.4953	0.4991	11.62	0.003
Lineal	2	2.4315	2.4315	1.2157	28.32	0.000
T (°C)	1	2.3579	2.3579	2.3579	54.92	0.000
t (h)	1	0.0736	0.0736	0.0736	1.71	0.232
Quadratic	2	0.0611	0.0611	0.0306	0.71	0.523
T (°C) * T (°C)	1	0.0011	0.0000	0.0000	0.00	0.998
t (h) * t (h)	1	0.0600	0.0600	0.0600	1.40	0.276
Interaction	1	0.0027	0.0027	0.0027	0.06	0.810
T (°C) * t (h)	1	0.0027	0.0027	0.0027	0.06	0.810
Residual error	7	0.3005	0.3005	0.0429		
Lack of fit	3	0.1282	0.1282	0.0427	0.99	0.482
Pure error	4	0.1724	0.1724	0.0431		
Total	12	2.7958				

Table S4. Analysis of variance for the recovery of phenolic compounds from PH by CSE using water–ethanol. Note: This model includes all the terms. The symbol "*" represents the interaction between variables.

Source	df	Sum of squares Seq.	Sum of squares Adjust.	Mean squares Adjust.	F	p
Regression	9	29.6089	29.6089	3.2899	10.64	0.000
Lineal	3	7.1044	1.9177	0.6392	2.07	0.168
Ethanol (%)	1	6.6434	1.7900	1.7900	5.79	0.037
T (°C)	1	0.4131	0.0046	0.0046	0.01	0.906
t (h)	1	0.0479	0.0258	0.0258	0.08	0.778
Quadratic	3	22.4222	22.4222	7.4741	24.18	0.000
Ethanol (%) * Ethanol (%)	1	22.1460	21.3182	21.3182	68.96	0.000
T (°C) * T (°C)	1	0.0007	0.0005	0.0005	0.00	0.968
t (h) * t (h)	1	0.2756	0.2756	0.2756	0.89	0.367
Interaction	3	0.0822	0.0822	0.0274	0.09	0.965
Ethanol (%) * T (°C)	1	0.0379	0.0379	0.0379	0.12	0.733
Ethanol (%) * t (h)	1	0.0027	0.0027	0.0027	0.01	0.927
T (°C) * t (h)	1	0.0416	0.0416	0.0416	0.13	0.721
Residual error	10	3.0912	3.0912	0.3091		
Lack of fit	5	3.0648	3.0648	0.6130	116.18	0.000
Pure error	5	0.0264	0.0264	0.0053		
Total	19	32.7001				

Table S5. Estimated regression coefficients for the recovery of phenolic compounds from PH by ASE using water. Note: This model includes all the terms. The symbol "*" represents the interaction between variables.

Term	Coefficient	Standard error coef.	T	p
Constant	3.23179	0.36798	8.783	0.000
T (°C)	0.03599	0.00510	7.062	0.000
t (min)	0.06417	0.02013	3.188	0.015
T (°C) * T (°C)	-0.00004	0.00002	-2.206	0.063
t (min) * t (min)	-0.00186	0.00043	-4.309	0.004
T (°C) * t (min)	-0.00005	0.00012	-0.387	0.710
S = 0.112244	PRESS=0.541681			
R-square = 99.19%	R-square (pred) = 95.06%	R-square (adj) = 98.62%		

Table S6. Estimated regression coefficients for the recovery of phenolic compounds from PH by ASE using water–ethanol. Note: This model includes all the terms. The symbol "*" represents the interaction between variables.

Term	Coefficient	Standard error coef.	T	p
Constant	2.72086	2.64123	1.030	0.327
Ethanol (%)	-0.01587	0.03425	-0.463	0.653
T (°C)	0.06419	0.03104	2.068	0.066
t (min)	-0.00272	0.12906	-0.021	0.984
Ethanol (%) * Ethanol (%)	-0.00039	0.00019	-2.004	0.073
T (°C) * T (°C)	-0.00019	0.00012	-1.644	0.131
t (min) * t (min)	-0.00032	0.00253	-0.127	0.902
Ethanol (%) * T (°C)	0.00021	0.00020	1.076	0.307
Ethanol (%) * t (min)	-0.00016	0.00097	-0.162	0.874
T (°C) * t (min)	0.00031	0.00074	0.420	0.683
S = 0.655596	PRESS=30.7024			
R-square = 91.06%	R-square (pred) = 36.14%	R-square (adj) = 83.01%		

Table S7. Analysis of variance for the recovery of phenolic compounds from PH by ASE using water. Note: This model includes all the terms. The symbol "*" represents the interaction between variables.

Source	df	Sum of squares Seq.	Sum of squares Adjust.	Mean squares Adjust.	F	p
Regression	5	10.8665	10.8665	2.1733	172.50	0.000
Lineal	2	10.5952	0.6360	0.3180	25.24	0.001
T (°C)	1	10.5942	0.6282	0.6282	49.87	0.000
t (min)	1	0.0009	0.1281	0.1281	10.17	0.015
Quadratic	2	0.2695	0.2695	0.1347	10.69	0.007
T (°C) * T (°C)	1	0.0355	0.0613	0.0613	4.87	0.063
t (min) * t (min)	1	0.2340	0.2340	0.2340	18.57	0.004
Interaction	1	0.0019	0.0019	0.0019	0.15	0.710
T (°C) * t (min)	1	0.0019	0.0019	0.0019	0.15	0.710
Residual error	7	0.0882	0.0882	0.0126		
Lack of fit	3	0.0738	0.0738	0.0184	3.84	0.149
Pure error	4	0.0144	0.0144	0.0048		
Total	12	10.9547				

Table S8. Analysis of variance for the recovery of phenolic compounds from PH by ASE using water–ethanol. Note: This model includes all the terms. The symbol "*" represents the interaction between variables.

Source	df	Sum of squares Seq.	Sum of squares Adjust.	Mean squares Adjust.	F	p
Regression	9	43.7765	43.7765	4.8641	11.32	0.000
Lineal	3	40.5223	2.4332	0.8111	1.89	0.196
Ethanol (%)	1	13.2272	0.0923	0.0923	0.21	0.653
T (°C)	1	27.0890	1.8375	1.8375	4.28	0.066
t (min)	1	0.2061	0.0002	0.0002	0.00	0.984
Quadratic	3	2.6695	2.6695	0.8898	2.07	0.168
Ethanol (%) * Ethanol (%)	1	1.5071	1.7268	1.7268	4.02	0.073
T (°C) * T (°C)	1	1.1555	1.1610	1.1610	2.70	0.131
t (min) * t (min)	1	0.0069	0.0069	0.0069	0.02	0.902
Interaction	3	0.5847	0.5847	0.1949	0.45	0.721
Ethanol (%) * T (°C)	1	0.4976	0.4976	0.4976	1.16	0.307
Ethanol (%) * t (min)	1	0.0113	0.0113	0.0113	0.03	0.874
T (°C) * t (min)	1	0.0758	0.0758	0.0758	0.18	0.683
Residual error	10	4.2981	4.2981	0.4298		
Lack of fit	5	4.0834	4.0834	0.8167	19.02	0.003
Pure error	5	0.2147	0.2147	0.0429		
Total	19	48.0746				
